# Molecular Aspects of a Diet as a New Pathway in the Prevention and Treatment of Alzheimer’s Disease

**DOI:** 10.3390/ijms241310751

**Published:** 2023-06-28

**Authors:** Julia Doroszkiewicz, Jan Mroczko, Piotr Rutkowski, Barbara Mroczko

**Affiliations:** 1 Department of Neurodegeneration Diagnostics, Medical University of Bialystok, 15-089 Bialystok, Poland; mjanek2003@gmail.com (J.M.); mroczko@umb.edu.pl (B.M.); 2 Staszica Mental Health Center, 15-071 Bialystok, Poland; p.rutk@mp.pl; 3 Department of Biochemical Diagnostics, Medical University of Białystok, 15-089 Bialystok, Poland

**Keywords:** Alzheimer’s disease, diet, nutrition, BDNF, mild cognitive impairment

## Abstract

Alzheimer’s disease is the most common cause of dementia in the world. Lack of an established pathology makes it difficult to develop suitable approaches and treatment for the disease. Besides known hallmarks, including amyloid β peptides cumulating in plaques and hyperphosphorylated tau forming NFTs, inflammation also plays an important role, with known connections to the diet. In AD, adhering to reasonable nutrition according to age-related principles is recommended. The diet should be high in neuroprotective foods, such as polyunsaturated fatty acids, antioxidants, and B vitamins. In addition, foods capable of rising BDNF should be considered because of the known profitable results of this molecule in AD. Adhering to beneficial diets might result in improvements in memory, cognition, and biomarkers and might even reduce the risk of developing AD. In this review, we discuss the effects of various diets, foods, and nutrients on brain health and possible connections to Alzheimer’s disease.

## 1. Alzheimer’s Disease

The most common cause of dementia in the world, Alzheimer’s disease (AD), puts an enormous burden on the healthcare system as a whole. Patients over the age of 65 are typically the ones who develop the illness. Every year, we see an increase in the number of impacted patients as a result of an aging society. By 2050, it is expected that there will be more than 100 million people worldwide suffering from AD dementia. It is a chronic disorder, since pathological processes begin at least 20 years before the onset of the disease. The hallmarks of AD are numerous, incompletely understood, and improperly categorized as part of the aging process. It is thought that the disease’s etiology may be influenced by both inherited and environmental factors. Despite the fact that several gene mutations are associated with AD, less than 5% of all cases are genetic. While early-onset AD (EOAD) is brought on by mutations in presenilin 1, presenilin 2, and amyloid precursor protein, sporadic, late-onset AD (LOAD) is associated with the APOE 4 gene (APP) [1]. The two main histological signs of AD are the gradual growth of neurofibrillary tangles (NFTs) formed of hyperphosphorylated tau and extracellular amyloid plaques. As a result, they cause loss of neurons and synapses [2].

As AD is currently incurable, preventative strategies are being actively discussed. The rates of clinical development of AD medications are currently limited, and the majority of medical research is focused on delaying progression rather than curing patients. It is because of the disease’s heterogeneity, underlying hallmarks, and pathogenesis, which are not yet completely understood. While there is currently no cure for Alzheimer’s disease, recent research has suggested that dietary interventions could help to prevent or delay the onset of disease.

## 2. Pathology of Alzheimer’s Disease

Amyloid buildup and tau protein hyperphosphorylation are the two primary pathogenic features of the illness. The aftereffects of these pathologic processes include neurodegeneration with loss of synapses and neurons, which results in macroscopical atrophy. Mixed pathology, which includes Lewy bodies and vascular disease, is a frequent occurrence, particularly in elderly adults [1]. The type 1 transmembrane protein known as amyloid precursor protein (APP) is expressed by a variety of cell types. Gamma- and beta-secretases in the central nervous system can successively cleave APP in two different ways [3]. The major components of incorrectly folded amyloid plaques, accumulating extracellularly, are Aβ40 and Aβ42, two products of APP metabolism. Aβ-42 is more prevalent than Aβ-40 inside plaques due to its higher incidence of fibrillization and insolubility. Aβ may then begin a series of processes that lead to synaptic loss and neuronal death by locating among healthy neurons and causing disruption in signaling, including neuroinflammation, and it is known that Aβ is capable of starting an immune response or creating angiopathy [4,5].

Neurons are built of a protein called tau, which assists in keeping the cytoskeleton’s microtubules intact in healthy cells [6]. It accumulates within nerve cell bodies as a result of the hyperphosphorylation that causes NFTs to tangle. Cellular proteins, which these tangles subsequently improperly engage with, are unable to carry out their typical functions [7]. A reduction in tau binding to microtubules leads to the malfunctioning of synapses. As a result of increased tau phosphorylation and intracellular tau aggregation brought on by an imbalance in tau kinase and phosphatase activity, NFTs are generated in AD patients. Last but not least, the growth of NFTs disrupts synaptic plasticity [7,8], which harms neuronal cells. According to research, Aβ accumulation may act as a trigger for the subsequent hyperphosphorylation process [9]. Moreover, there is evidence that toxic tau can boost Aβ synthesis via a feedback loop mechanism [10].

**Figure 1:**
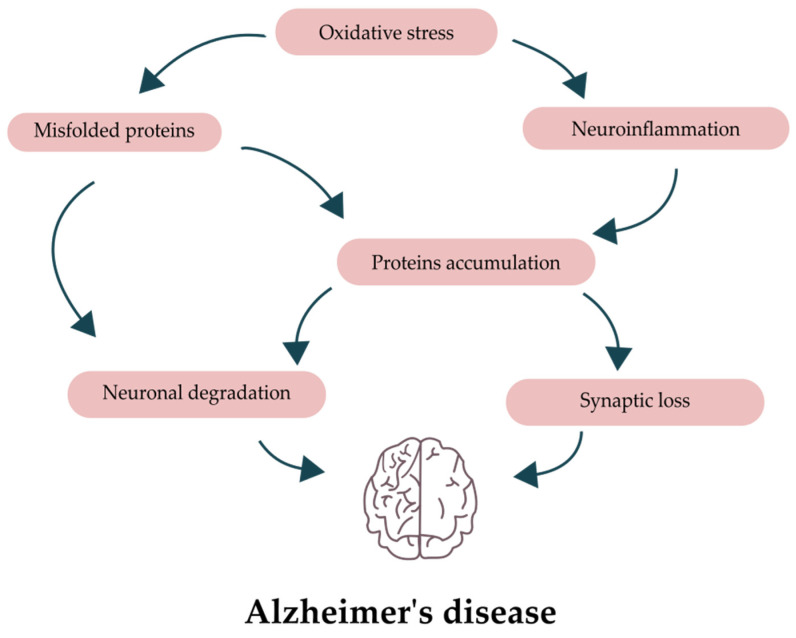
Mechanisms underlying Alzheimer’s disease pathology.

Neuroinflammation is a newly identified characteristic that is receiving a lot of attention. Even after more than 20 years of research, the processes of AD neuroinflammation are still not fully known. The two most important components of the inflammatory response in the brain are microglia and astroglia. Microglia can be of two kinds, which can be activated: M1 and M2. The M1 phenotype is regarded as “pro-inflammatory” and conventional, whereas the M2 phenotype is “anti-inflammatory” and unconventional [11]. Classical activation is brought on by the release of pro-inflammatory molecules such as IL-1, TNF, IL-6, and reactive oxygen species and is associated with pathogen defense mechanisms [12]. The M2 phenotype, which is induced by IL-4 and IL-13, on the other hand, releases neuroprotective agents such as TGF-β, IL-10, and IGF-1 [12]. By controlling inflammation, M2 microglia can help improve tissue remodeling and repair. It is noteworthy how rapidly the M1-to-M2 conversion can occur [13,14]. Moreover, senile plaque growth is promoted by the pro-inflammatory environment that active microglia surrounding the plaques generate [15,16]. These mechanisms are depicted in the Figure 1.

On the other hand, the neurotrophic factors are the growth factors promoting neuronal growth, differentiation, survival, and proper functioning in mature and developing nervous systems [17]. They interact with one of two receptors: p75 neurotrophin receptor (p75NTR), a member of the tumor necrosis factor (TNF) receptor superfamily, or tyrosine receptor kinase (Trk), one of the tropomyosin-related tyrosine kinase receptors. Neurotrophins (NGF binds to TrkA; BDNF and NT4/5 bind to TrkB; and NT3 binds to TrkC), which interact with certain Trk receptors, promote survival and growth responses. Additionally, their interaction with the P75 neurotrophic receptor (p75NTR) affects both apoptosis and brain plasticity [17]. Brain-derived neurotrophic factor (BDNF) is the most abundant neurotrophin and is responsible for promoting neuroprotection and neuroregeneration. This well-researched growth factor in the mammalian brain plays an important role in maintaining synaptic plasticity in learning and memory [18,19]. The majority of BDNF produced in the brain is delivered to presynaptic terminals and postsynaptic dendrites from the cell bodies of neurons and glial cells. The release of neurotransmitters, ion channel activity, axonal pathfinding, and neuronal excitability are all regulated by BDNF and its receptor, tropomyosin receptor kinase B (TrkB), which is localized at glutamate synapses [20]. Apoptosis, neuroinflammation, tau phosphorylation, and Aβ buildup are all linked to BDNF deficiency in the etiology of AD [21]. With the activation of TrkB and phosphatidylinositol 3-kinase (PI3K) signaling, BDNF stimulation causes tau to dephosphorylate [22]. By deregulating the glutamatergic N-methyl-d-aspartate receptor (NMDAR)/Ca^2+^/calpain signaling cascade, Aβ impairs BDNF function [23]. The extracellularly regulated kinase/cyclic AMP response element-binding protein (ERK/CREB) signaling pathway can increase BDNF, which can minimize the impact of Aβ on dendritic atrophy and neuronal death [24]. Increasing evidence also indicates the significance of BDNF signaling in regulating the long-term effects of Aβ buildup in AD. By controlling the neurotransmitters that are released (including glutamate and gamma-aminobutyric acid) after nuclear factor B (NF-B) activation, BDNF controls the relationship between inflammation and neuroplasticity [25]. Reduced BDNF levels were found in AD patients’ brains, blood, and cerebrospinal fluid [26,27,28] as the illness advanced. Moreover, increased cognitive performance in AD has been associated with elevated serum BDNF levels [18,29]. These results have raised interest in BDNF as a prospective biomarker for AD diagnosis or as treatment.

## 3. Diets and Life Situations Negatively Influencing the Disease

### 3.1. Obesity and Malnutrition

There is no dispute about the fact that obesity, one of the risk factors for AD, is connected to low-grade chronic inflammation. In fact, people with obesity or other metabolic illnesses have approximately a twofold-increased chance of getting AD, according to meta-analysis research [30,31]. Consuming a high-fat diet, which is abundant in high saturated fatty acids, has been linked to obesity but also to deficiencies and hippocampus-dependent learning and memory function [32,33]. Rats fed a high-fat diet depicted impaired memory, which progressed with further administration of the diet [34]. In addition, prospective epidemiological studies support the connection between high-fat diet and dementia [35]. Elevated levels of APP in adipose tissue and Aβ in plasma are two potential culprits in the development of AD in obese people. As indicated by Lee et al.’s research, increased adipocyte APP gene expression may be the cause of mid-life obesity-related increases in Aβ plasma levels. Increased transport of Aβ into the human brain may occur as a result of chronic elevation in Aβ plasma levels [31,36]. Furthermore, adipocytes and monocyte-derived macrophages in adipose tissue secrete acute-phase proteins, such as CRP, and pro-inflammatory cytokines, such as IL-6, IL-1, and TNF-alfa, which contribute to neuroinflammation [37]. Chronically consuming too many high-carbohydrate and high-saturated-fat meals while obese can have a negative influence on insulin production, which in response has a major impact on brain glucose metabolism and functioning. Loss of or diminished insulin sensitivity is one of the symptoms of insulin resistance. Reduced glycogen storage in muscle and fat cells and a decline in hepatic glucose absorption are both results of peripheral insulin resistance [31]. Peripheral insulin resistance triggers CNS insulin signaling to worsen, which then causes changes in cerebral metabolism. In fact, high insulin levels or peripheral insulin resistance lower insulin transporter levels and lower BBB permeability to insulin [38]. One of the variables underpinning the pathophysiology of AD has been identified as changes in insulin signaling in the brain [39]. According to this theory, several AD models and AD patients have decreased brain insulin receptor sensitivity, which may indicate the development of amyloid plaques in the brain.

On the other hand, in patients with AD, especially in those with moderate to severe AD, nutritional issues, particularly weight loss, are commonly observed. Moreover, it is a reliable indicator of worse outcomes in individuals suffering from the condition [40,41]. Recent research revealed that mild cognitive impairment (MCI) and early-stage AD are precursors to nutritional issues, such as appetite changes, weight loss, and sarcopenia [41,42,43]. Moreover, a prior study revealed that a low body mass index (BMI) indicates a higher risk of dementia after MCI [44]. Malnutrition issues appear to be significant though controllable aspects that may alter the prognosis of dementia, despite the fact that the precise processes behind nutritional issues in AD patients are not fully known. The biggest detrimental effects on brain function and mortality in individuals with cognitive impairment are undernutrition and weight loss, which accelerate the neurodegenerative process. In severe dementia, Hanson et al. have shown that weight loss is a predictor of mortality [45]. The most prevalent finding is that low BMI and malnutrition are associated with an increased risk of dementia and death, demonstrating the role of nutrition in the neurodegenerative process [46]. Furthermore, in patients with mild AD, malnutrition was associated with disease progression [47]. According to Yu et al., adults over 65 with a trend toward weight reduction should have their cognitive function constantly examined [48].

### 3.2. Diet High in Fats

Dietary fat can be divided into saturated and unsaturated dietary fats. Monounsaturated fatty acids (MUFAs) and polyunsaturated fatty acids (PUFAs), which include omega-3 fatty acids, are two categories of unsaturated fats. While MUFAs and PUFAs (“good fats”) are assumed to have positive benefits, including enhanced brain function and the avoidance of neurodegenerative diseases, saturated and trans fats (“bad fats”) are widely recognized to have deleterious impacts on human health, including correlations to AD. Unsaturated fats that have undergone partial hydrogenation, or trans fats, are used to lengthen the food shelf life span [49,50]. Evidence from studies on animals supports the harmful effects of saturated and trans fats on pathology and behavior related to AD. In a mouse model of AD (5xFAD), ingestion of a diet heavy in saturated and trans fats was enough to increase cerebrovascular Aβ deposition and hippocampus oxidative stress and promote cognitive impairment [51]. Furthermore, in an AD transgenic mouse model (APP/PS1), consumption of a diet high in fat was linked to memory impairment, a rise in Aβ monomers and plaques, and brain inflammation [50,52]. These revelations were confirmed by epidemiological studies according to which consuming saturated and trans fats increases the chance of developing Alzheimer’s disease and dementia [53,54]. A more recent meta-analysis thoroughly assessed four independent prospective cohort studies and depicted that high consumption of saturated fats is also strongly linked to an elevated risk of AD (39%) and dementia (105%) [55]. Interestingly, dietary lipids can affect how the CNS is in an inflammatory state. Microglia respond with inflammation to saturated fatty acids by secreting pro-inflammatory cytokines [56,57].

### 3.3. Western Diet

The phrase “Western diet” (WD) refers to a modern eating style that is typical to Western nations. It is based on ultra-processed foods that are ready for consumption; created using refined materials; and are high in salt, simple sugars, saturated fatty acids (SFAs), and cholesterol. Grain, fiber, and mono- and polyunsaturated fatty acids (MUFAs and PUFAs; particularly anti-inflammatory omega-3, -6, and -9 acids) are also insufficient in this dietary pattern. Moreover, the WD has a major negative impact on how the gut and its commensal bacteria operate, which indirectly reduces the absorption of minerals and vitamins from the diet [58,59]. There is a significant amount of proof that the WD can worsen cognition, learning, and memory in mice and humans, as well as aggravate or cause the pathogenic aspects of AD [60,61,62,63]. Two dietary elements, fat and sugar, were shown to have the most significant influence on hippocampal processes. When mice were fed a diet high in SFAs, sucrose/sugar, or both, for short or long periods, there were a variety of deficits in hippocampus-dependent learning and memory behavioral tests, which was comparable with evidence from individuals who ate following a diet high in SFAs for three days [64,65,66]. In addition, the WD is linked to a reduced total volume of the left hippocampus, which was described in research performed on 60- to 64-year-old men [59,67]. Additionally, research performed on animals confirmed the effects of the WD on the amyloidogenic accumulation of toxic Aβ peptides and tau phosphorylation. Overall, mice fed a Western diet showed a significant rise in typical biomarkers of AD, such as Aβ, Aβ plaques, tau, and ptau [68,69,70]. Moreover, biomarkers of inflammation such as TNF-alfa, IL-6, or IL-1beta were also enhanced in animals fed the WD [65,71,72]. These revelations support the negative influence of the WD on patients. Table 1 summarizes the effects of diets and life situations negatively influencing the disease.

## 4. Diets with Positive Influence

### 4.1. Mediterranean Diet

The Mediterranean diet (MeDi or MD) is a nutritional style that is popular in nations bordering the Mediterranean Sea, such as Greece or southern Italy. The traditional Mediterranean diet is characterized by high intake of fruits, vegetables, whole grains, nuts, and legumes; moderate intake of fish, poultry, and alcohol (especially red wine, with meals); and low intake of red and processed meats, with olive oil being the primary source of fat [73,74]. Considering that it contains all the necessary nutrients, such as monounsaturated fatty acids (primarily found in olive oil), polyunsaturated fatty acids (found in fatty fish), antioxidants (such as carotenoids, beta-carotene flavonoids, indoles, or lutein), vitamins (A, B types, D, and E), and minerals (calcium, iodine, magnesium, potassium, selenium, and zinc), it can be used as a nutritional model for healthy eating habits [75,76]. Many chronic illnesses have been linked to a preventive impact of the MeDi pattern.

Higher MD adherence has been linked to improved cognitive function [77,78], a slower pace of cognitive deterioration [79,80], and a decreased risk of cognitive impairment [81,82] but also AD itself [83,84]. Longitudinal research described by Anastaiou et al. depicted several connections between individual components of the MeDi and cognition [85]. For example, there was a reduction in the risk of dementia of 68.9% after every serving of fish per day [85]. The study by Ballarini et al. showed that lower Aβ42/40 ratio and pTau pathologies were observed in patients with higher adherence to a MeDi [86]. A newer randomized trial described the effects of the diet in mid-life on Alzheimer’s biomarkers. In cognitively normal people, Mediterranean-like diets improved cerebral blood flow; memory; and AD biomarkers, such as lowered Aβ40 levels and improved Aβ42/40 ratio. Compared with cognitively normal people, adults with MCI had an unusual pattern, with the Western diet having a positive impact on cerebrospinal fluid biomarkers and the MeDi elevating t-tau levels [87]. Interestingly, increasing the MeDi score, which describes adherence to the diet, in a study on Greek patients resulted in men with decreased risk of cognitive impairment but elevated risk in the women’s group [81].

### 4.2. DASH Diet

The DASH diet, which is similar to the Mediterranean diet in that it requires a substantial intake of plant-based foods, also restricts the consumption of SFAs, total fats, cholesterol, and salt. The DASH eating pattern encourages greater consumption of preventive nutrients such as K, Ca, Mg, fiber, and vegetable proteins while simultaneously encouraging lower intake of refined carbs and saturated fats [88]. It has been demonstrated that the DASH dietary pattern, which was created to prevent and treat hypertension, improves risk factors for cardiovascular disease (CVD), such as total cholesterol, and systolic and diastolic blood pressure [88,89].

Several studies examined the effects of the DASH diet on AD. In older adults with cognitive impairment without dementia (CIND), better verbal memory was present while adhering to the diet [90]. However, many studies describe no significant results of the DASH diet on AD [91,92,93].

### 4.3. MIND Diet

The Mediterranean–DASH Intervention for Neurodegenerative Delay diet, a cross between the Mediterranean and DASH diets, was created to stress nutrients linked to preventing dementia and to avoid components, such as saturated/hydrogenated fats, that have been linked to dementia [94]. The MIND diet suggests eating more than or equal to three servings of whole grains per day, which is the core of the diet [95]. The next important component of the diet is eating more than or equal to six servings of leafy green vegetables per week (in addition to one or more daily servings of other vegetables) [96]. Moreover, it also entails consuming more than or equal to two servings of berries per week [97], more than or equal to one serving of fish per week [98], more than or equal to two servings of poultry per week, more than three servings of beans per week, and more than or equal to five servings of nuts per week [99]. The MIND diet encourages the use of olive oil as the main source of fat [100] and permits one serving of wine or alcohol per day [94,101]. Moreover, this diet should not be based on red meat or processed food such as fast food and should avoid frying [94]. According to several prospective cohort studies, eating more vegetables was linked to a slower rate of cognitive decline, with green leafy vegetables showing the highest associations [96,102,103]. Due to its dietary elements, which have antioxidative, anti-inflammatory, and neuroprotective properties, the MIND diet may support brain health. Hosking et al.’s research indicated that the MIND diet was linked to a lower risk of 12-year cognitive impairment and that stronger adherence to the diet was linked to a 53% lower risk of impairment [103]. In models of adjusted logistic regression, the MIND diet was linked to a lower risk of cognitive deterioration over the course of 12 years [103]. Moreover, a decreased rate of cognitive impairment following stroke was linked to high MIND diet adherence [94].

### 4.4. Other Diets

#### 4.4.1. Ketogenic Diet

Ketogenic diet (KD) refers to a high-fat, medium-protein, and low-carbohydrate diet that leads to a metabolic shift to ketosis [104]. Carbohydrates should be reduced to less than 10% of the diet, which triggers the shift from glucose metabolism to metabolizing fatty acids [105]. In neurodegenerative diseases, there is evidence that a ketogenic diet (KD) and/or exogenous ketone supplementation may be helpful in the treatment of AD patients [106]. A complicated interplay of metabolism, gut microbiome, and other mechanisms can regulate neuroinflammation in neurodegenerative diseases by activating multiple molecular and cellular pathways. Recent and accumulating studies on human and animal models have shown that the KD is beneficial in neurodegenerative diseases by modulating central and peripheral metabolism, mitochondrial function, inflammation, oxidative stress, autophagy, and the gut microbiome [107]. The effect of a ketogenic diet on the inhibition of AD development is multidirectional; the KD prevents chronic sleep deprivation (SD)-induced AD by inhibiting ferroptosis and improving the neuronal repair ability via the Sirt1/Nrf2 signaling pathway [108]. In the study by Xu et al., four months of KD improved spatial learning, spatial memory, and working memory in mice [109]. The authors observed an improvement in cognitive functions, which was associated with a restored number of neurons and synapses in both the hippocampus and the cortex. Ketogenic diet treatment also decreased amyloid plaque deposition and microglial activation, resulting in reduced neuroinflammation [109]. In clinical studies, the KD was associated with better cognition but also improved brain metabolism and AD biomarkers, with lowered tau concentrations and increased Aβ42 cerebrospinal fluid levels in patients with MCI [110]. Currently, it seems that the KD can provide therapeutic benefits to patients with neurological problems by effectively controlling the balance between pro- and antioxidant processes, and between pro-excitatory and inhibitory neurotransmitters and by modulating inflammation [111]. It is a common phenomenon that energy deprivation in neurological disorders, including Alzheimer’s disease, progresses rapidly. The ability of ketone bodies to stabilize mitochondrial energy metabolism makes them suitable intervening agents [112]. The ketogenic diet is a good candidate for adjuvant therapy, but its specific applicability depends on the type and the degree of the disease [104].

#### 4.4.2. Fasting

Given the many health advantages of intermittent fasting (IF), it has lately grown in popularity, also considering its positive effects on AD. According to studies on the regulation of metabolic pathways, intermittent fasting (IF) has been shown to increase the life span and prevent or postpone the onset of age-related disorders. Improvements in glucose tolerance, lipid metabolism, and cognitive impairment are just a handful of the positive consequences of IF [113]. When examining the molecular processes, fasting has a significant impact on the neurochemistry and activity of the neural network, particularly in certain parts of the brain, such as brainstem, striatum, hypothalamus, and hippocampus. Several signaling pathways have been shown to play a role in the structural and functional adaptations of neuronal circuits to nutritional limitation, particularly low glucose and amino acid levels, including enhanced synaptic density and neurogenesis [75,114].

There are two types of IF: alternate day fasting (ADF) and time-restricted feeding (TRF). ADF involves 24 h of dietary restriction every other day, followed by a day of unlimited feeding. With no limitation on the quantity of food or nutrients consumed, TRF restricts daily meal consumption to 8 h or fewer. Animal experiments have also used other forms of IF, such as prolonged fasting (fasting for two or more days), the 5:2 strategy (2 fasting days interspersed with 5 days of regular feeding), and time-controlled fasting (40 h of fasting, 24 h of feeding, followed by 24 h of fasting and 80 h of feeding in each cycle) [113,115].

Although TRF is a more known type of food restriction diet and mainly used to lose weight, ADF has more positive outcomes relatively to AD. Clinical research has mainly concentrated on the improvement of ADF in obese individuals, which includes lowering weight and total cholesterol, promoting fat oxidation, and reducing insulin resistance [116,117]. In individuals who are normal weight or overweight, ADF is also helpful for cardioprotection as it decreases triacylglycerol concentrations and increases low-density lipoprotein (LDL) particle size [118]. In regards to body weight and heart disease, greater results were observed with ADF combined with exercise [119]. In the case of neurodegeneration, ADF is frequently used in mice models of neurodegeneration and aging. ADF improved synaptic plasticity, and learning and memory deficiencies in elderly mice by upregulating the expression of synaptophysin (Syn) and protein kinase CaMkinase (CaM) [120]. However, ADF affected mice models in different ways. ADF decreased the excessive amyloid buildup in the APP/PS1 animal model of AD, though it had no effect on amyloid deposition in 3xTg AD [121,122]. Furthermore, ADF enhanced neuroinflammatory responses in female 5xFAD, increased GABA signaling, and decreased neuronal function [123]. Nevertheless, after ADF intervention, the survival rate, spatial learning, and memory abilities in the AppNL-G-F mice model greatly improved, with no notable change in Aβ accumulation [124]. Moreover, in another mice model, IF has been linked to improvements in brain structure, such as a thicker CA1 pyramidal cell layer and greater expression of the dendritic protein drebrin in the hippocampus, as well as decreased oxidative stress [125].

**Figure 2:**
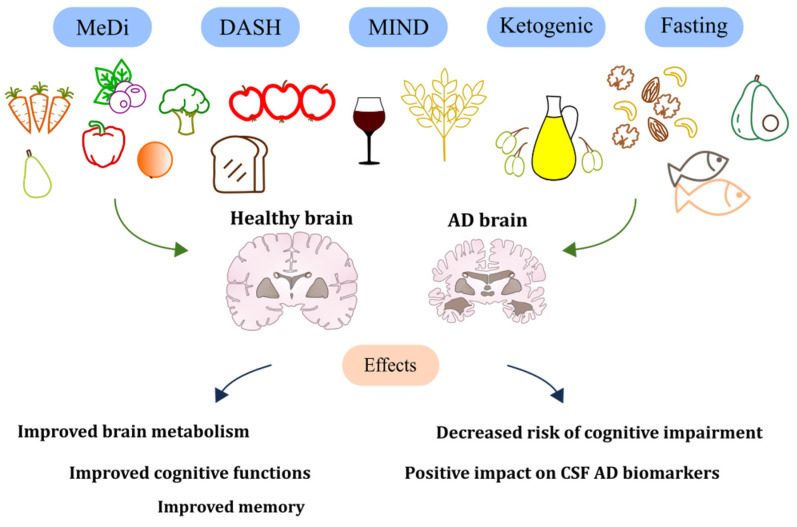
Illustration of the positive effects of diets.

In human studies so far, several researchers have highlighted the possible benefit of protein restriction against the aging process and aging-related chronic disorders. At this time, no research has been performed on protein or calorie restriction in human individuals diagnosed with AD [75,126]. It is difficult to imagine that severe food restrictions could be sustained for extended periods, particularly among elderly subjects with neurodegenerative diseases. However, short periods of caloric restriction were described as capable to enhance cognitive functions such as verbal memory, and 30 days of low-glycemic-index diet in patients with MCI resulted in the advancement of delayed visual memory and the CSF biomarker Aβ42 [127,128]. Positive results of the above-described diets are presented in Figure 2.

## 5. Beneficial Foods and Nutrients

For a long time, a wide range of human illnesses, including cancer, cardiovascular disease, respiratory diseases, infectious diseases, diabetes, obesity, metabolic syndromes, and neurological problems, have been successfully treated with natural remedies based on plants and herbs. These products have also been used to slow down the aging process. The majority of diets with numerous cell-protective properties, such as anti-inflammatory, antioxidant, and anti-arthritis properties, and capable of improving memory and cognitive abilities come from natural sources. Therefore, we aim to summarize the beneficial aspects of foods and nutrients with a known connection to AD.

### 5.1. Curcumin

Curcumin is a polyphenol found in turmeric and a spice with a unique yellow color that is commonly used in cooking. Due to its rich therapeutic qualities, turmeric has drawn a lot of interest among many natural medicines [129]. Curcumin may have anti-amyloid effects on AD, according to many lines of evidence. Firstly, curcumin inhibits Aβ aggregation as well as disaggregation to create fibrillar Aβ40 according to research performed in vitro [130]. Numerous in vivo investigations described that it promotes the disaggregation of existing amyloid deposits, prevents the formation of new ones, and even reduces the size of surviving deposits [131,132]. Moreover, curcumin and its derivatives suppress the synthesis of fibrillar Aβ from Aβ monomers and destabilize preformed fibrillar Aβ in vitro, showing that curcumin is protective against Aβ toxicity [133]. According to existing theories, curcumin works primarily by inhibiting NFkB, which is accomplished by preventing IkB phosphorylation and subsequent NFkB activation [134]. Curcumin can also prevent APP-cleaving enzymes such as B-secretase from functioning (BACE-1) [135,136].

Although animal studies show promising results, research performed on humans is limited and not trustworthy, which impedes the understanding of the results [137,138,139]. Only a modest number of clinical research studies have discussed the impact of curcumin on human cognitive performance, with conflicting results. While several research studies claim that curcumin has no beneficial effects on cognitive function [140,141], others show valuable results in protecting against cognitive decline [137,138]. Furthermore, neuroimaging demonstrates that curcumin could decrease Aβ brain deposits [137].

### 5.2. Coffee and Tea

Coffee is a beverage that contains a great amount of polyphenols. Moreover, it is known that caffeine increases human information processing, attentiveness, and reaction time [142]. The positive outcomes of caffeine are connected to antagonizing A1 adenosine receptors in the hippocampus and cortex [143]. Caffeine has well-known anti-inflammatory characteristics that include reducing the invasion of immune cells from the periphery, attenuating pro-inflammatory mediators, and inhibiting microglia activation. Greater plasma caffeine concentrations were associated with lower levels of inflammatory cytokines in the hippocampal region [56,144]. Animal studies imply that coffee with caffeine has a crucial function, since it lowers plasma Aβ levels, as opposed to caffeine-free coffee [145]. A meta-analysis by Wu et al. has shown that a diminished risk of development of dementias in humans (including AD) was observed with daily consumption of one–two cups of coffee [146]. Interestingly, research part of the Italian Longitudinal Study on Aging (ILSA) suggested that older people with normal cognitive function who increased their coffee consumption had a higher rate of developing MCI, while constant-over-time, moderate coffee consumption was linked to a lower rate of MCI incidence [147,148]. However, research does not support the theory that caffeine could prevent AD [149], although as reported in epidemiological research, drinking coffee and caffeine can possibly help treat AD [147].

One of the most popular beverages worldwide is tea, as it is the most consumed, second only to water. It is a byproduct of the plant *Camellia sinensis* (L.) O. Kuntze. Regardless of how much fermentation has occurred, green tea, black tea, and oolong tea are all produced from the same plant [150,151]. The health advantages of green tea have been the subject of research the most, including its effects on diabetes, cancer, cardiovascular diseases, and neurological disorders [152,153]. It mainly contains polyphenols. Despite the fact that the mechanisms behind green tea’s preventative impact are not fully understood, there are some hypotheses. It has been shown that oxidative stress has a role in the etiology of both AD and vascular dementia (VaD) [154,155]. Firstly, green tea catechins working in the brain have antioxidant properties with known strong free radical scavenger abilities [156]. Then, green tea polyphenols have an anti-inflammatory effect via the inhibition of nuclear factor kappa-beta activation [157]. Interestingly, epigallocatechin gallate (EGCG), the main component of green tea catechins, has shown inhibitory properties against Aβ accumulation [154,158]. Mice models of AD fed EGCG for weeks depicted an indicative decrease in Aβ aggregation and lowered neurotoxicity associated with amyloid [159]. Moreover, based on research by Tomata et al., drinking green tea frequently was substantially linked with having a low risk of dementia [160]. In a rat model of Alzheimer’s disease, green tea was found to improve memory and prevent oxidative stress and damage to the hippocampus [161].

### 5.3. Cocoa

Theobroma cocoa beans, which are often consumed by humans, are used to make cocoa (powder) and chocolate. Such products are abundant in flavonoids, the greatest category of dietary polyphenols and antioxidant compounds [162,163]. Cocoa beans are reported to have more antioxidant activity than green tea, red wine, or blueberries [164]. The polyphenol content in cocoa and chocolate is not the only difference between them; cocoa is a ground-up form of cocoa beans, whereas chocolate is a mixture of cocoa, cocoa butter, sugar, and other components that are combined to create a solid food product [165]. Chocolate has varying amounts of cocoa, which vary from 30–70% in dark chocolate to 7–15% in milk chocolate [166]. Additionally, the bioavailability of cocoa flavanols tends to be regulated by the complexity of the food matrix; therefore, consuming dark chocolate may result in less effective absorption of these chemicals than ingesting cocoa powder [163]. Human studies have demonstrated that consuming a particular combination of flavanols in cocoa can lower blood pressure, enhance insulin sensitivity, and improve cerebral perfusion and blood flow [167,168,169]. Research on the effects of cocoa in the rat pheochromocytoma PC12 cell line treated with Aβ(25–35) has shown that cocoa flavonoids have neuroprotective effects, preventing Aβ-induced cell death [170,171]. Interestingly, the findings of mouse models show that cocoa extracts may successfully stop the oligomerization of Aβ [172]. These revelations prompted researchers to conduct the study called CoCoA Study (Cocoa, Cognition, and Aging). They investigated the possibility that dietary flavanols might enhance cognitive performance in participants with MCI [173]. Desideri et al. showed that consuming a beverage fortified with cocoa flavanols (CFs) improved cognitive functions in adults diagnosed with MCI and lowered blood pressure and insulin resistance [173]. The results were confirmed by a randomized controlled trial showing comparable outcomes [168].

### 5.4. Vitamins

It was observed that patients’ cognitive abilities can be enhanced by having them consume a diet high in vitamins, especially those with antioxidant properties. Oxidative stress causes the reduction of O_2_ to H_2_O in mitochondria, which promotes the production of reactive oxygen species (ROS). As a consequence of their high reactivity, they interact with many molecules and structures. All macromolecules are observed to be oxidized quite early in AD patients’ brains. In AD, oxidation occurs in lipids, proteins, nucleic acids, and polysaccharides. Therefore, antioxidants such as vitamins are widely discussed as beneficial in AD [174]. Vitamins from the B family vary in terms of functions and structure. Folates play a significant role in the etiology of neurological illnesses by providing the methyl groups required for DNA methylation [175,176]. Homocysteine by itself is related to increases in cardiovascular risk and cognitive impairment. The metabolism of homocysteine is linked to the positive effect of vitamin B on cognitive performance. There is a reduction in DNA methylation in AD patients, and insufficiency of these vitamins leads to a buildup of homocysteine in the body that affects the genes connected to AD with their overexpression [176]. Therefore, researchers studied the supplementation of vitamin B, and the results showed decreased concentrations of homocysteine with simultaneous better results in MMSE, and semantic and episodic memory [177]. The results were tested in AD patients with similar results while supplementing folic acid [178]. However, the results are not consistent, with several publications not documenting a significant impact on cognitive decline or even documenting no connection [179,180]. In conclusion, the examination of individual articles demonstrates that the impact of nutritional intervention in the form of dietary supplementation with vitamins B appears to be ambiguous. High blood levels of vitamin B12 may be a protective factor, even if it has been noted that testing for the presence of folate and vitamin B12 is unreliable [175].

High frequency of vitamin D hypovitaminosis, particularly in the elderly, is caused by dietary habits and inadequate sun exposure. According to recent studies, more than 50% of central Europeans over 60 may not be getting sufficient amounts of vitamin D. Vitamin D supplementation seems to be especially beneficial for this sensitive age group, since vitamin D hypovitaminosis is linked to numerous disorders, including AD [181,182,183]. It is described that it can double the risk of dementia and increase the risk of developing AD by 21% [184,185]. Ghahremani et al. showed that when compared with no exposure, dementia incidence was 40% lower when vitamin D was provided [186]. Moreover, the benefits of vitamin D were much stronger in women than in men and in those with normal cognition than in people with mild cognitive impairment, and apolipoprotein E4 non-carriers had much stronger vitamin D effects than carriers [186]. Several research studies found changed plasma Aβ1-40 levels, slower development of AD symptomatology, and lowered Aβ-related biomarkers following vitamin D treatment, which would indicate a lower risk of AD [187,188].

Vitamin E is a fat-soluble vitamin that shows neuroprotective and anti-inflammatory properties [189,190]. Therefore, researchers have considered it for potential therapeutic usage in AD. A meta-analysis by Lopes da Silva et al. showed lower plasma levels of vitamin E in AD patients in comparison to cognitively healthy controls; moreover, they came to the conclusion that the patients’ malnutrition was not the cause of this result [191]. This was also confirmed by a newer and bigger study by Mullan et al. [192]. Additionally, a number of prospective cohort studies examined plasma vitamin E levels and the probability of developing AD, with results showing significant correlation with higher levels of vitamin E and reduced risk of cognitive impairment or even reduced risk of developing AD [193,194]. Thus, supplementing this vitamin was also studied, with interesting evidence. Greater dietary vitamin E intake was linked to a reduced risk of AD, although the effect was only shown in people who did not have the ApoE4 risk allele [195,196]. Interestingly, the same results were not obtained when supplementation of this vitamin was non-dietary [195]. In randomized trials, supplementing α-tocopherol alone led to a slower functional decline compared with the placebo group. Furthermore, this study also showed that the combination of memantine and α-tocopherol was not as beneficial as α-tocopherol alone [197]. However, many studies show no changes in patients’ behavior, biomarkers, or cognitive decline; therefore, the connection between vitamin E and treating AD is still being discussed [198,199].

### 5.5. Foods Rising BDNF

As described above, BDNF is an interesting factor playing a role in the brain also in AD. Specifically altering patients’ diet may be a useful strategy for influencing BDNF and maintaining or even enhancing cognitive and metabolic health. Thus, numerous intervention trials with the goal of increasing BDNF levels have been conducted as a result of the fact that higher levels are linked to better cognitive function [200]. Although various research studies have shown no significant increase in BDNF in connection with dietary interventions, there are some with interesting outcomes [117,201]. Suzuki et al. reported that in female MCI patients, consuming mold-fermented cheese significantly increased serum BDNF levels as compared with non-mold-fermented cheese administration [202]. In healthy subjects, intake of kernel-based whole grain (WG) rye products was described as capable to increase BDNF concentrations [203]. In addition, zinc supplementation had beneficial effects on women with premenstrual syndrome and obese ones [204,205]. Moreover, curcumin, further to the above-described beneficial effects on AD itself, was also described as influencing BDNF levels in the plasma of depressed patients [206]. However, this area needs more research, especially in connection to AD.

## 6. Summary

Overall, there is growing evidence that the molecular aspects of a diet can play an important role in the prevention and treatment of Alzheimer’s disease. By consuming a diet that is rich in omega-3 fatty acids, antioxidants, and plant-based foods, individuals may be able to reduce their risk of developing Alzheimer’s disease and slow the progression of the disease if it does occur. In our article, we discuss the potential of a specific diet and diet interventions to prevent and treat Alzheimer’s disease. We reviewed the literature on the role of diet in Alzheimer’s disease and propose new aspects for preventing and treating the disease based on the molecular aspects of a specific diet. Research performed on diets rich in fruits, vegetables, whole grains, legumes, nuts, fish, and olive oil suggests that diet can modify the molecular pathways involved in Alzheimer’s disease by reducing inflammation, oxidative stress, and insulin resistance. We also discuss diets and life situations negatively influencing the disease. Research shows that both obesity and malnutrition have a significantly bad influence on the course of AD or result in a higher chance of developing the disease. Moreover, diets high in fat and sugar intake contribute to higher levels of AD and inflammatory biomarkers. This review suggests an important role of food intake in the course of the disease and suggests potential dietary intervention in the treatment of AD.

## Figures and Tables

**Table 1:** The effects of diets and life situations negatively influencing the Alzheimer’s disease.

Authors	Type of Diet/Life Situation	Study Design	Study Population	Results	Reference
Lee et al.	Obesity	Pilot study	10 obese patients before and after weight loss intervention	Changes in adipocyte APP expression correlated with changes in plasma Aβ40 levels (R = 0.74, *p* = 0.01).	[36]
Ye et al.	Malnutrition	Longitudinal study	747 patients with aMCI	The underweight group had a higher risk of probable Alzheimer’s disease dementia (pADD), and the decreased-BMI (HR: 2.29, 95% CI: 1.41–3.72) groups were at increased risk of progression to pADD.	[44]
Hanson et al.	Malnutrition	Prospective cohort	256 nursing home residents with advanced dementia and feeding problems	Significant mortality risk in patients with feeding problems	[45]
Laitinen et al.	High-fat diet	Population-based study	1449 patients with 117 who had dementia	Moderate intake of polyunsaturated fats in mid-life decreased the risk of dementia even after adjustment for demographic variables, especially among the ApoE epsilon4 carriers.	[54]
Ruan et al.	High-fat diet	Meta-analysis of cohort studies	8630 participants and 633 cases	A higher dietary saturated fat intake was significantly associated with increased risks of 39% and 105% for AD and dementia.	[55]
Jacka et al.	Western diet	Longitudinal study	255 persons from the Personality and Total Health Through Life Study	Higher consumption of an unhealthy “Western” dietary pattern was associated with smaller left hippocampal volume.	[67]
Gibson et al.	Western diet	Observational study	23 women with polycystic ovary syndrome from 25 to 45 years old	Greater intakes of saturated and trans fats, and higher saturated-to-unsaturated fat ratio (Sat:UFA) were associated with more errors in the visuospatial task and poorer word recall and recognition.	[62]

## Data Availability

Not applicable.
